# “All they do is walk”: Successful aging and symbolic boundaries among a self-organized mall walkers club

**DOI:** 10.1093/geroni/igab046.2877

**Published:** 2021-12-17

**Authors:** Jason Pagaduan

**Affiliations:** University of Toronto, Scarborough, Ontario, Canada

## Abstract

Objectives: This study examines how successful aging discourse manifests through physical and social participation among members of a self-organized mall walkers club. There is a paucity of research investigating successful aging in situ and theorizing the relationship between successful aging discourse and community participation. I draw on symbolic boundaries—a concept from cultural sociology—as a way to make sense of what mall walkers say and do. Methods: I draw on data from 15 months of participant observations and interviews of mall walkers, all of whom are over 65 and predominantly Caribbean-Canadian women Results: I identify three common boundaries: personal, interpersonal, and community, that mall walkers draw on to challenge narratives of decline and internalize dimensions of successful aging. Discussion: These findings uncover the ways members in a self-organized community reinforce boundaries that highlight how certain dimensions of successful aging as something to be proud of and desirable. This article contributes to research on intersubjective experiences of aging by revealing how successful aging is rooted in community participation, rather than individual achievement.

